# Panoramic errors in pediatric patients with special needs

**DOI:** 10.1038/s41598-023-38823-1

**Published:** 2023-07-20

**Authors:** Avia Fux-Noy, Rasha Rohana, Andra Rettman, Moti Moskovitz, Chen Nadler

**Affiliations:** 1 Department of Pediatric Dentistry, Hadassah Medical Center, Faculty of Dental Medicine, Hebrew University of Jerusalem, P.O.B. 12272, 9112102 Jerusalem, Israel; 2 Oral Maxillofacial Imaging Unit, Oral Medicine, Sedation and Maxillofacial Imaging, Hadassah Medical Center, Faculty of Dental Medicine, Hebrew University of Jerusalem, Jerusalem, Israel

**Keywords:** Dental radiology, Paediatric dentistry, Special care dentistry

## Abstract

This study aimed to analyze the types and frequencies of panoramic technical errors in pediatric patients with special needs, as compared to matching images of patients with normal developmental patterns. Panoramic images of 100 pediatric patients, with equal numbers of either special needs or healthy controls, referred to our Oral Maxillofacial Imaging unit, were retrospectively examined by four blinded observers for the presence of four common technical errors: palatoglossal air space, movement, positioning errors, and foreign bodies appearance. In addition, they subjectively determined the image quality on an ordinal scale. The statistical examination included inter-observer correlation and correlations between demographic factors (age, gender, developmental status) and the number and types of errors. The frequencies of demonstrated errors were, in descending order: movement, positioning, palatoglossal air space, and foreign bodies' appearance. The special needs group images showed significantly more errors and were rated as low-quality radiographs. Younger patients in both groups showed more movement and positioning errors. Technical errors in panoramic images of patients with special needs were more frequently found. Therefore, increased awareness of the staff and appropriate pre-imaging instructions to the patients, are required when imaging pediatric patients, especially those with special needs.

## Introduction

Radiographic imaging serves as a valuable diagnostic tool in pediatric dentistry. Diagnostic radiology is aimed at obtaining images that address the clinical query while exposing the patient to low doses of radiation. Images should be diagnostic to ensure optimal patient care, since low-quality images may lead to an incorrect diagnosis, followed by improper treatment. In addition, non-diagnostic images may dictate repeated imaging, which may increase the patient’s radiation dose and increases the risk of cancer, especially in pediatric patients^1,2^.

Panoramic radiography gives a two-dimensional image that provides an overview of the jaws and their surrounding structures. It is widely used in pediatric dentistry, due to its ability to capture the entire dentition in a single, extra-oral image with a relatively low patient radiation dose. Indications of pediatric panoramic radiography are (1) growth and development evaluation and teeth location, shape, and position assessment; (2) craniofacial trauma; (3) impacted third molar; (4) bone lesions and cysts; (5) temporomandibular joint (TMJ) disorders evaluation; and (6) identification of foreign bodies^3,4^.

Improper patients posture and positioning, artifacts appearance due to foreign bodies, improper positioning of the tongue against the palate which results in the demonstration of the palatoglossal air space (PAS), and patient movement during exposure time are some of the factors which reduce image quality; these factors can be divided into technical-related errors and inadequate patient/parent cooperation^5,6^.

Imaging the pediatric patient can be challenging, especially for children with special needs. The imaging process can spark fear and anxiety thus causing inadequate patient cooperation. Effective communication between staff members, the child, and the accompanying family member is of great importance. It is necessary to adjust the instructions given by the staff to the child's age, level of cognitive development, and medical condition^6–10^. The American Academy of Paediatric Dentistry has defined children with special needs as children who suffer “any physical, developmental, mental, sensory, behavioral, cognitive, or emotional impairment or limiting condition that requires medical management, health care intervention, and/or use of specialized services or programs. The condition may be congenital, developmental, or acquired through disease, trauma, or environmental cause and may impose limitations in performing daily self-maintenance activities or substantial limitations in a major life activity”^11^. Developmental delays (DD) occur when the child does not achieve developmental milestones as compared to children in the same age range. The delay may involve speech and language, motor, social, and/or cognitive development^12^. Caring for these children requires special knowledge, increased awareness and attention, adaptations, and special means beyond what is considered routine.

Currently, no study has examined the effect of a child's developmental state on the quality of panoramic images. It is reasonable to assume that panoramic images of patients with developmental delays will have more errors as compared to images of children with normal development (ND) patterns, and thus when children with DDs need to undergo panoramic imaging they will require specific accommodations. The present study aims to compare the types and frequencies of panoramic errors in pediatric patients with DDs, as compared with those of ND patterns.

## Results

In this retrospective study, four observers, blinded to the clinical data, examined 50 DD and 50 ND panoramic images for the presentation of image errors and overall quality. The two groups were age and gender matching, mean age of each group was 12.06 ± 3.8 years, and 70% were males. The distribution of different types of DD are presented in Table 1.

**Table 1:** Distribution of differt types of DD.

DD type	No. of patients
Autism	14
Down syndrome	9
Developmental delay	5
Cerebral palsy	3
Intellectual disability	3
Williams syndrome with cognitive impairment	2
Hydrocephalus and developmental delay	2
Angelman syndrome	2
Epilepsy, language impairment and developmental delay	1
Ectodermal dysplasia and developmental delay	1
Osteopetrosis and blindness	1
Agenesis of the corpus callosum	1
Myelodysplastic syndrome and developmental delay	1
Noonan syndrome and intellectual disability	1
Paralysis due to car accident	1
Pierre Robin syndrome with cognitive impairment	1
Joubert syndrome	1
Congenital myopathy	1
Total	50

The mean kappa value for the inter-examiner agreement was 0.95 (95% CI 0.84–0.90) (Table 2).

**Table 2:** Kappa value for the inter-examiner agreement.

	Kappa value	95% CI	p-value
PAS appearance	0.94	0.86–1.02	0.00
PAS involved side	0.96	0.84–1.01	0.00
Movement appearance	0.94	0.86–1.02	0.00
Number of movements appearance	0.99	0.91–1.07	0.00
Horizontal plane errors	0.95	0.86–1.05	0.00
Superimposition of the spine (anterior-posterior error)	0.98	0.90–1.06	0.00
Foreign body	0.95	0.87–1.03	0.00
Image quality	0.92	0.84–1.02	0.00

**Figure 1:**
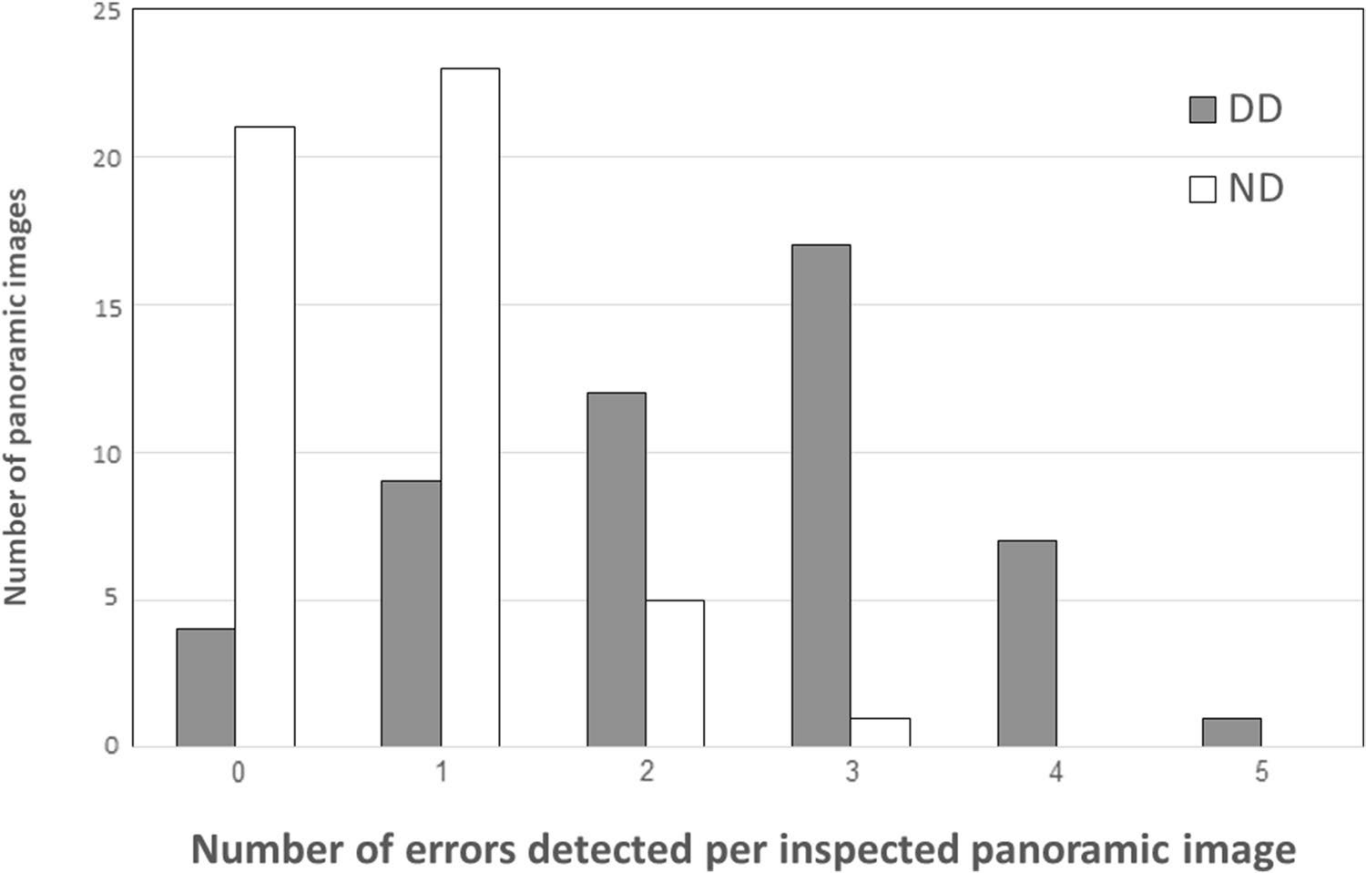
Number of errors detected per inspected panoramic image.

**Figure 2:**
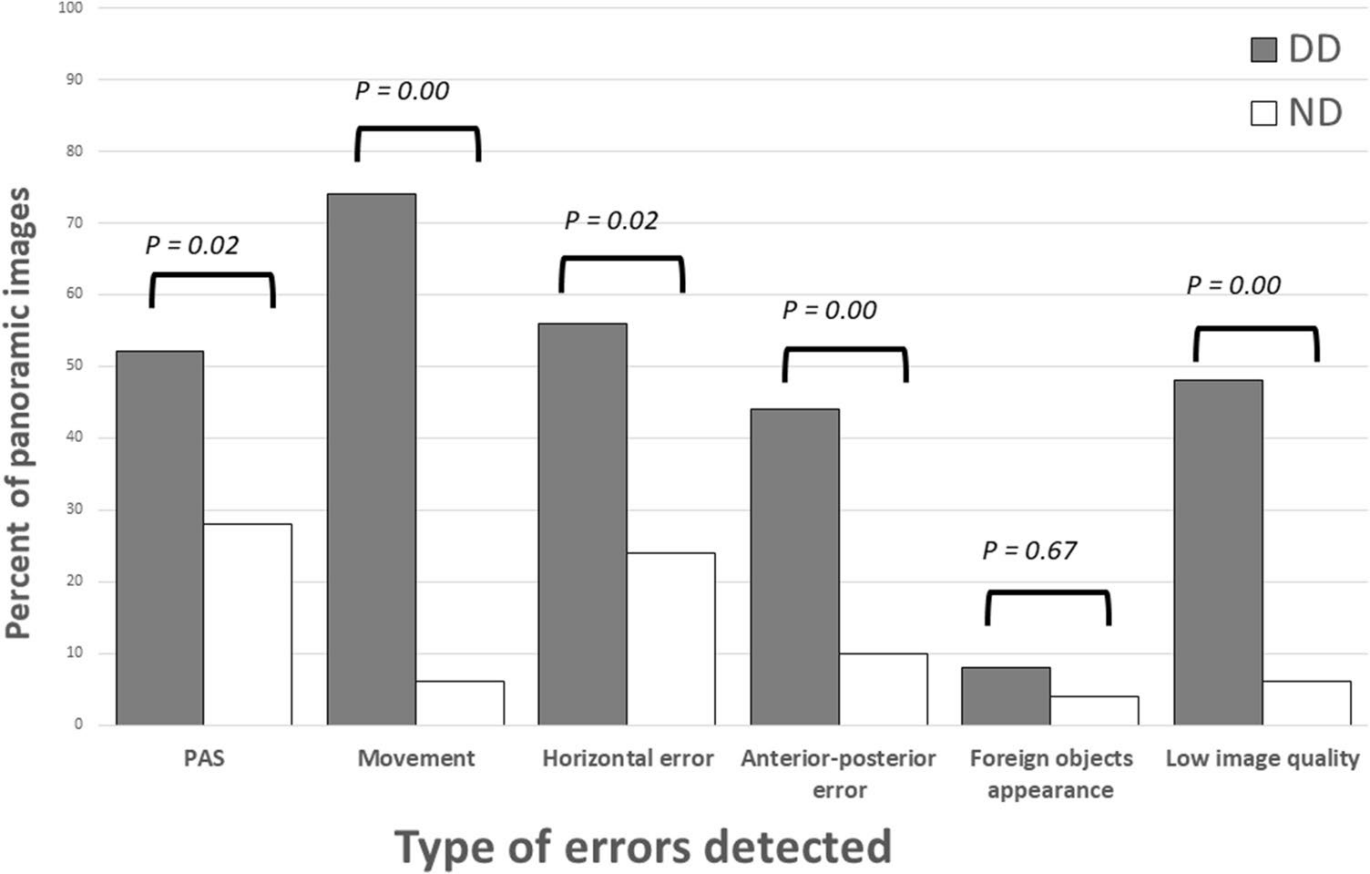
The distribution of different errors and image quality in the DD group as compared with the ND group.

The distribution of number of errors per radiograph in the DD group as compared with the ND group is shown in Fig. 1. Almost half of the images of the ND group (n = 21) were error free as compared to only four in the DD group. The distribution of different errors and image quality is shown in Fig. 2. The frequency of errors was significantly higher for every error type in the DD group (PAS appearance p = 0.024, movement appearance p = 0.000, horizontal plane errors p = 0.002, superimposition of the spine p = 0.000), except for the foreign body category (p = 0.67), with movement being the most frequent error found in the DD group (Table 3). Panoramic radiographs of the DD group were significantly rated as radiographs with low quality (p = 0.00).

**Table 3:** The distribution of different errors and image quality according to developmental status (DD compared to ND) and gender (boys compared to girls).

	ND, n (%)	DD, n (%)	p-value	Boys, n (%)	Girls, n (%)	p-value
PAS appearance	14 (28)	26 (52)	0.024	34 (48.6)	6 (20)	0.008
Movement appearance	3 (6)	37 (74)	0.000	28 (40)	12 (40)	0.9
Horizontal plane errors	12 (24)	28 (56)	0.002	28 (40)	12 (40)	0.9
Superimposition of the spine	5 (10)	22 (44)	0.000	20 (28.6)	7 (23.3)	0.63
Foreign body	2 (4)	4 (8)	0.67	4 (5.7)	2 (6.7)	0.92
Low image quality	3 (6)	24 (48)	0.00	21 (30)	6 (20)	0.33

No statistically significant difference was found between genders except for the frequency of PAS, that was significantly higher in boys (p = 0.008) (Table 3).

When analysing the relationship between age and panoramic errors, age had a strong negative correlation with two technical errors evaluated: movement error and positioning error (horizontal and AP). Thus, the younger the child, the more likely it was to find movement and positioning errors (horizontal and anterior-posterior(AP)) (Table 4).

**Table 4:** The correlation between age and each variable.

	Correlation coefficient value (*r*)	*p v*alue
Error type		
PAS	0.066	0.53
Movement	–0.209	0.037
Horizontal error	–0.35	0.00
AP error	–0.26	0.009
Foreign objects	0.21	0.035*
Low image quality	0.23	0.016*

*The significant relationship disappeared in multivariate logistic regression analysis.

Logistic regression found that PAS appearance was associated with gender (p = 0.01) and developmental status (p = 0.012), movement appearance was associated with age (p = 0.007) and developmental status (p = 0.000), horizontal plane errors were associated with age (p = 0.00) and developmental status (p = 0.001), and superimposition of the spine was associated with age (p = 0.005) and developmental status (p = 0.000) (Table 5).

**Table 5:** Logistic regression for the effect of the independent variables (age, gender and developemental status) on radiogragh errors.

		Sig	Exp(B)	95% C.I.for EXP(B)	
				Lower	Upper
PAS	Age	0.475	1.043	0.930	1.169
	Gender	0.010	3.979	1.391	11.381
	ND/DD	0.012	0.327	0.137	0.784
Movements	Age	0.007	0.766	0.633	0.929
	Gender	0.685	1.309	0.356	4.819
	ND/DD	0.000	0.011	0.002	0.060
Horizontal plane	Age	0.000	0.783	0.686	0.893
	Gender	0.610	1.303	0.471	3.600
	ND/DD	0.001	0.187	0.071	0.498
Superimposition of the spine	Age	0.005	0.821	0.715	0.942
	Gender	0.335	1.759	0.559	5.539
	ND/DD	0.000	0.113	0.035	0.364
Foreign body	Age	0.065	1.477	0.976	2.235
	Gender	0.575	0.588	0.092	3.763
	ND/DD	0.394	0.459	0.076	2.757
Low image quality	Age	0.052	1.150	1.006	1.314
	Gender	0.436	1.523	0.528	4.389
	ND/DD	0.488	1.382	0.554	3.444

## Discussion

This study aimed to analyse common panoramic errors among children with DD as compared with those of ND pattern. Our results showed errors in both groups, with more panoramic errors (especially movement and positioning errors) in DD population as compared to ND matching population, which was inversely correlated with age. Overall, only 25% of the evaluated panoramic radiographs were error-free, and in patients with DD only 8% were error-free.

Intraoral radiographs may be uncomfortable for pediatric patients, especially if the patients have DD, sometimes to the point it is impossible to perform the radiographs. In some cases, a panoramic image can be used instead of an intra-oral radiograph. However, panoramic imaging requires 8.2–19.0 s^13^, which compared with the shorter time for intraoral radiographs, increases the risk of being affected by movement or lack of correct positioning during the scan. Studies indicate that panoramic radiography is prone to various errors and image quality which fundamentally depend on correct positioning and cooperation of the patient^4–6,14,15^.

This study is novel as it focuses on errors in patients with special needs. Granlund et al. and Peretz et al. investigated errors in panoramic images of healthy pediatric patients with mixed dentition and found that the incidences of error free images were 4% and 2.7%, respectively^6,14^. Furthermore, both Granlund et al. and Peretz et al. found that PAS appearance was the most common error, appearing in 79% and 64% of panoramic images, respectively. In the current study we found the same frequency for PAS, movement, and positioning errors (80%)^6,14^. The frequencies were significantly higher in the DD group as compared to the ND group. Images of younger patients showed more movement and positioning errors. Movement during exposure was the most frequent error found among the study group (74% as compared to only 6% in the ND group). Anxiety-provoking experiences and a lack of cognitive abilities to follow instructions might explain this finding^9,10,16,17^.

In this study more than half of the images (56%) were considered low-quality images, 48% in the DD group as compared to only 6% in the ND group. This finding was consistent with other studies. Peretz et al.^14^ found 45% of the radiographs of patients with mixed dentition, and 39% of the radiographs of patients with permanent dentition, were of low quality. In addition, Choi et al.^18^ investigated the level of clinical image quality of panoramic radiographs among children and adults and found 41% of the images were of poor quality.

The differences in errors' frequency and image quality between the current study and the other studies^6,14,18^ may be attributed to the fact that panoramic images taken in our imaging unit are performed by three experienced technicians with more than 10 years of experience. It is possible that in other imaging centers the frequencies of errors will be much higher, maybe to a point where it will be impossible to image these children at all, which will impair the possibility of providing them with appropriate treatment. Alternatively, patients with special needs may be treated with compromised images, thus their treatment may be compromised as well.

Children with special needs are a widespread population. In 2015, the Israel National Council for the Child reported that approximately 13% of Israeli children are with special needs^19^, and there is an increasing prevalence of developmental difficulties among Israeli children^20^. Imaging these children can be challenging to the patient, parent, and technician due to lack of cooperation, poor technician-patient/parent communication skills, technician knowledge gaps on the issue, and body/skeletal malformations that might be present^21,22^. Therefore, it is appropriate to develop protocols for radiographic examination suitable for this population. Dailey and Brooks^23^ provided guidelines for applying basic and advanced techniques for dental radiographic examinations for children with autism spectrum disorder. These techniques included proper preparation, visual schedule, visual incentives, social stories, and the use of sedation. There is a need for more similar studies that include various diagnoses and special needs.

One limitation of the study is that it includes a sample of radiographs available in the medical records. It is possible that there were patients in whom a panoramic radiograph was not performed due to the patient's inability to position himself in the machine properly or without moving. The data about the non-performance of panoramic for these reasons were not documented or difficult to locate, which leads to missing information regarding the extent of the population not capable of performing panoramic radiographs due to DD. Another limitation is that DD comprises a wide spectrum of conditions with an extensive array of clinical manifestations. This makes the test group heterogeneous, making it difficult to draw specific conclusions or guidelines.

In conclusion, our study suggests that the imaging of young pediatric patients, especially those with DD, must be performed in special suitable centers with special equipment and protocols, to have diagnostic images. Knowledgeable and skilled staff is needed to position the child in an appropriate position, maintain the position during exposure, and more importantly to achieve diagnostic imaging for effective treatment planning with reduced repetitions. This would decrease the risk of radiation exposure, as pediatric patients are more radiosensitive than adults.

## Methods

In order to retrieve the relevant panoramic images for our study, we reviewed all panoramic images conducted at the Oral Maxillofacial Imaging unit, during January 2019- December 2021.

From the retrieved panoramic images our study group comprised 50 panoramic radiographs of patients under 18 years with DDs, mostly including children diagnosed with congenital or acquired physical and or intellectual disabilities, such as Down syndrome, autism spectrum disorders, cerebral palsy, as specified in Table 1. The control group comprised 50 radiographs of matching age and gender patients, with ND pattern. Exclusion criteria were panoramic images of patients with other, non-developmental systemic diseases such as bleeding disorder, diabetes etc. or panoramic images included in our medical records, which were taken outside our institute.

In case there were more than one image per patient for the same date (retakes), we included the first non-diagnostic panoramic image, for either the study or the control group. All radiographs were taken by three experienced personnel with more than 10 years of experience using the ORTHOPANTOMOGRAPH^®^ OP200 D machine, (Instrumentarium Dental Inc., Tuusula, Finland). The DICOM files of the panoramic images were retrieved and anonymized and stored on a special folder on ON-DEMAND 3D software (cybermed) on a flat panel liquid crystal display (HP Display Z23i 23-inch IPS LED Blacklit monitor) with a high-definition graphic processing unit (ATI Radeon HD 2400 Series; 8 Advanced Micro Devices).

Anonymized images from both groups were randomized and presented to four observers (two oral medicine specialists and two pediatric dentistry specialists) blinded to the clinical data. The observers were asked, for each radiograph, to mark the presence/absence of four specific errors (specified below) as well as subjectively assess the image quality as either high quality or low quality.

Furthermore, a pre-evaluation calibration session by all observers, using five panoramic images with different errors, not included in the study was done, prior to dataset assessment.

**Figure 3:**
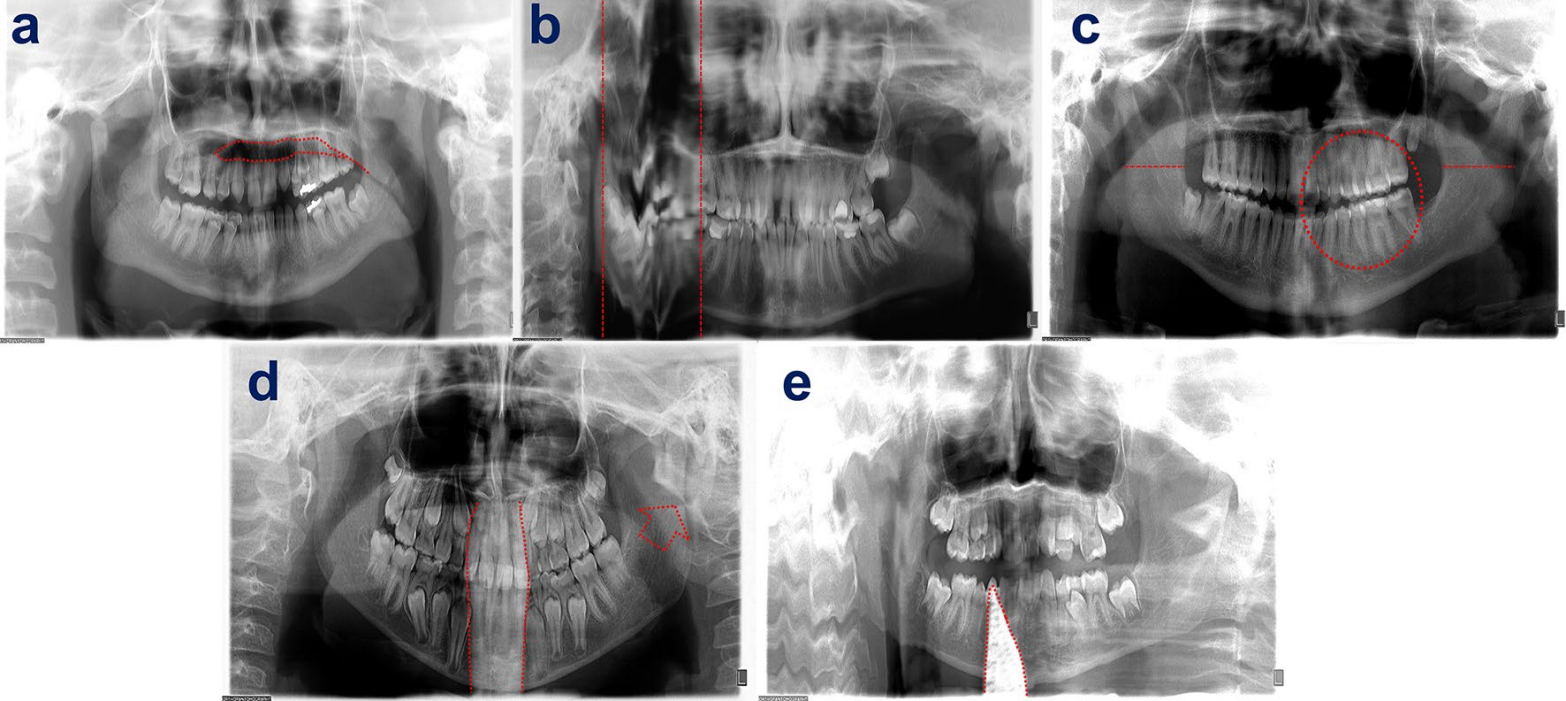
Examples of panoramic technical errors: (**a**) palatoglossal airway superimposed on the maxilla. (**b**) Schematic presentation of distortion due to movement at the right side of the panoramic image indicated by two horizontal red lines. (**c**) Horizontal misalignment causing asymmetrical structures (red lines) and the overlapping proximal surfaces (red circle). (**d**) Anterior–posterior misalignment causing demonstration of the vertebral ghost image (two curved red lines) as well as vertebral superimposition on left ramus (red arrow). (**e**) Lead apron artifact on top of the anterior mandible (red lines) and distortion due to movement on the right side.

The four common panoramic errors examined were^4–6,14^ (Fig. 3):

1. Palatoglossal air space (PAS) appearance: PAS is the oral cavity air shadow between the tongue and the hard palate which can obscure periapical pathology in the maxillary teeth. To reduce PAS, it is necessary to instruct the child to attach the dorsal part of the tongue to the palate throughout the imaging process.
2. Movement: Panoramic exposure time is between 8.2 and 19.0 s^13^. Movement of the child during this time will form typical anatomical effects by stretching or refracting the image, leading to a "fracture" appearance of the jaw^15^.
3. Positioning errors (PE): Patient's Frankfort plane should be parallel to the ground. To obtain a sharp image, the jaw’s basal part must be positioned correctly during the rotational movement of the device to ensure the Frankfort plane is parallel to the horizontal plane, the midsagittal plane is perpendicular to the horizontal plane, and there is a proper anterior–posterior (AP) plane. If the midsagittal plane of the patient is not correctly aligned the structures imaged becomes asymmetrical and proximal areas of crown surfaces become substantially overlapped (horizontal error). If patient’s head is positioned either too far forward or too far backwards it will cause ghost images appearance and superimposition of anatomical structures (AP error).
4. Artifacts’ appearance due to foreign objects.

The study was approved by the Institutional Human Subjects Ethics Committee (HMO-0279–20), which waived patient's informed consent.

Data was tabulated to Excel and processed using SPSS software (statistical package for the social sciences), version 25.0 (SPSS, Inc., Chicago, Illinois, USA). Examiners' reliability was measured according to the Fleiss’ Kappa test. When there was disagreement between the observers, the majority rule was used to create a unified answer. The mean and standard deviation (SD) were used to summarize and describe continuous variables. Categorical variables were described by frequencies and percentages. In the preliminary stage of data processing, the differences between the study groups in terms of socio-demographic variables and background information were examined using the ANOVA test and the Chi-squared test. Chi-squared test was used to compare the two study groups. The strength of the association between a continuous variable and a categorical variable was tested according to Spearman's correlation coefficient. Logistic regression estimated the quality of the integrated model for each of the indices, and ANOVA tests examined the differences between the different age groups. The significance level was set to p < 0.05.

### Compliance with ethical standards

The study was approved by the committee on research involving human subject of the Hebrew University- Hadassah Medical School, Jerusalem, Israel (HMO-0279-20), which certified that the study was performed in accordance with the ethical standards as laid down in the 1964 Declaration of Helsinki and its later amendments or comparable ethical standards.

## Data Availability

The datasets generated during and/or analyzed during the current study are available from the corresponding author on reasonable request.
